# Frequency of Structural Brain Abnormalities among Adult Diagnosed with Attention – Deficit/Hyperactivity Disorder

**DOI:** 10.5539/gjhs.v6n1p69

**Published:** 2013-10-14

**Authors:** Nour-Mohammad Bakhshani, Samaneh Babaei, Mahvash Raghibi

**Affiliations:** 1 Children and Adolescent Health Research Center, Department of Clinical Psychology, Zahedan University of Medical Sciences, Zahedan, Iran; 2 Zahedan University of Medical Sciences, Zahedan, Iran; 3 Department of Psychology, Faculty of Education and Psychology, Sistan and Baluchestan University, Zahedan, Iran

**Keywords:** attention deficit disorder with hyperactivity, hyperkinetic syndrome, magnetic resonance imaging, attention

## Abstract

Although ADHD is known as a childhood disorder, it is prevalent among adults as well. Several studies have been conducted on the etiology of this disorder and its neurobiological and neuroanatomical manifestations in children, but the knowledge of adult ADHD is not enough. The present research was aimed at studying the structural brain abnormalities in adult ADHD cases. Fifteen adult patients diagnosed with ADHD, developed during their childhood, were selected for this study. In addition to clinical interview and Magnetic Resonance Imaging (MRI), all the participants were asked to fill the (ASRS-VI.I). The results indicated that about 40 % of adults with ADHD suffer from structural brain abnormalities. The results of MRI showed that 100% of the individuals with inattentive and combined types of ADHD were structurally damaged but MRI results did not reveal any structural brain abnormalities in hyperactive participants. The results of the present study are somewhat consistent with the results of previous studies. In general, any brain injury in the region related to cognitive processes (such as attention, memorization, and prevention) and brain circuits related to motor functions and motivation can contribute a role to the induction of ADHD symptoms. It is recommended to conduct more researches in the future with larger samples using other methods that are capable of assessing brain performance and the level and mechanism of the functions of neurotransmitters and neuronal modulators.

## 1. Introduction

Attention–Deficit/Hyperactivity Disorder (ADHD) is a common neuropsychological disorder worldwide. It first described as a childhood disorder that affects about 2-18% of school aged children (Faraone, Sergeant, Gillberg, & Biederman, 2003; Polanczyk et al., 2007; Rowland, Lesesne, & Abramowitz, 2002). Although it was first believed as a child-aged disorder but evidence showed that it continue into adulthood (Bakhshani, Babaei, & Raghibi, 2012; Kessler et al., 2006), and about 5% of adults may suffer from it (American Psychiatric Association, 2000; Biederman & Faraone, 2005; Faraone, Biederman, & Mick, 2006; Kessler et al., 2006). ADHD characterized by inappropriate levels of inattention, hyperactivity and impulsivity (American Psychiatric Association, 2000), that was initially known as hyperactive (hyperkinetic) disorder (Curatolo, D'Agati, & Moavero, 2010). Diagnostic Statistical Manual of Mental Disorders (DSM) proposed three subtypes of ADHD, including inattentive, hyperactive-impulsive and combined types (American Psychiatric Association, 2000). The Age, sex and stage of development may influence the clinical feature of ADHD, for example inattentive type is more common in females and aggressive or impulsive behaviors are prominent in hyperactive- impulsive type; while impairment in function and severity of symptoms are highest in combined type. A key clinical feature observed in patients with ADHD is comorbidity with other psychological problems. Low self-esteem, drug abuse and problem in relations with peers are prevalent in adolescents with ADHD (Elkins, McGue, & Iacono, 2007; Loe & Feldman, 2007). Antisocial behavior and substance abuse, anxiety and mood disorders are more among adults with ADHD (Secnik, Swensen, & Lage, 2005).

ADHD has a complex etiology and considering three subtypes of ADHD by APA (American Psychiatric Association, 2000), helps the researchers to organize their etiological hypotheses of ADHD around the central symptoms of each subtype (Castellanos et al., 2002; Nigg & Casey, 2005; Sergeant et al., 2003). Recently, findings provided by neuroimaging methods have demonstrated deficits in several regions of brain involved in response inhibition, attention and executive functioning (Curatolo, 2005; Curatolo et al., 2010; Krain & Castellanos, 2006; Suskauer et al., 2008). Prefrontal cortex, basal ganglia, cerebellum, the corpus callosum, gray and white matter of brain have been the specific areas in studies related to structural and neuroanatomy of ADHD (Curatolo et al., 2010; Krain & Castellanos, 2006). Various studies using structural magnetic resonance imagining in children with ADHD compared to controls have found abnormalities in some brain regions of ADHD patients, including parieto-temporal areas, the basal ganglia, posterior cingulate (PCC), the cerebellum and the splenium of the corpus callosum (Batty et al., 2010; Carmona et al., 2009; Cortese, 2012; Krain & Castellanos, 2006; Mackie et al., 2007; Rubia, 2011; Shaw et al., 2006). These regions mediate cognitive control functions. The largest volume reduction was in frontal lobes, right cerebral hemisphere, total cerebral volume, caudate and splenium of corpus callousum (Valera et al., 2007). These regions have important role on cognitive control functions. Frodl and Skokauskas performed a meta-analysis of MRI voxel-based morphometry (VBM) and manual tracing studies to identify the differences between adults and children with ADHD as well as between treated and untreated individuals. They found Basal ganglia regions are structurally affected in children with ADHD; and alterations in limbic regions are more pronounced in non-treated patients and seem to diminish over time from child to adulthood. Treatment of ADHD produces positive effects on brain structure (Frodl & Skokauskas, 2012).

Considering the core symptoms of ADHD, prefrontal brain regions and connections to the striatum may be most important regions involved in ADHD (Giedd et al., 2001; Stefanatos & Wasserstein, 2001). Although recently attempts have been done to examine neurobiological substrates of ADHD but the studies mostly have focused on childhood and little is known about neurobiological correlates of adult ADHD. However, about 30-60% of patients diagnosed with ADHD in childhood and adolescence generally continue to have symptoms into adulthood. Recently, efforts have been made to assess the neurobiological infrastructure of ADHD but these studies mainly have focused on child ADHD rather than adult ADHD. Therefore, the knowledge of the neuroanatomy of adult ADHD is inadequate and reports on medical images of the brain structure of adult ADHD cases are very scarce.

## 2. Patients and Methods

In this study, 15 adults (9 female and 6 male, mean age = 27.6, SD = 10.2) with pure ADHD recruited from the outpatients of psychiatric clinics in Zahedan and Sari (Iran). ADHD was diagnosed according to the diagnostic criteria of DSM-IV (American Psychiatric Association, 2000). All participants had a previous diagnosis of ADHD and treatment history using Ritalin from childhood. Patients participated in current study with full awareness and consent. In order to assess the severity of the symptoms, all the participants were completed the Adult ADHD Self Report Scale (ASRS-V1.1) (Kessler et al., 2005) and, also, were clinically interviewed. The ASRS-V1.1 was developed by the WHO and the work group on Adult ADHD. ASRS-V1.1 includes 18 questions, in two parts: Part A and Part B consisted with DSM-IV criteria. All participants asked to complete both Part A (6 questions) and Part B (12 questions, If 4 or more questions of part A marked in, the participant has symptoms of Adult ADHD). Part B provides more information related to frequency and severity of ADHD (Adler, Kessler, & Spencer, 2003). Sensivity, Specifity and predictive value of the ASRS-V1.1 are 68.7%, 99.5% and 89.3% respectively, and has internal consistency (Cronbach α), “between” 0.63 to 0.70. Also its reliability ranges “between” 0.58 to 0.7 (Kessler et al., 2007).

In addition, for diagnosing and locating possible brain abnormalities the participants were scanned by magnetic resonance imagining (MRI). MRI is a suitable tool for diagnosing different types of structural brain abnormalities such as tumor, abscess, infection, lesion, and brain and brain stem diseases. MRI is noninvasive and sensitive Neuro-imaging technique for examining abnormality of brain structure without using ionizing radiation. MRI provides high quality images using magnetic field and radio waves (Walsh & Darby, 1999). In this study we used MRI System with Magnetom Avanto Model.

## 3. Results

The results of the present study indicate of 15 participants with adult ADHD, 4 show symptoms of attention deficit, 9 show symptoms of hyperactivity/impulsivity, and 2 have combined type symptoms (Table 1). Based on the results obtained by MRI, about 40% (6 out of 15) of the participants suffer from at least one brain abnormality that is traceable by MRI. The areas with abnormalities are shown in the Table 2.

**Table 1:** Distribution of symptoms by types of ADHD among participants

Types of ADHD	**n**	**(%)**
Attention deficit Disorder	4	26.7
Hyperactivity Disorder	9	60
Combine	2	13.3
Total	15	100

As seen in Table 2, 100% of the participants with AD and combined types of ADHD suffer from one brain abnormality. However, brain MRI findings did not show structural brain abnormality in Hyperactive/Impulsive type.

**Table 2:** Frequency of brain abnormality based on ADHD type, age and gender

	Types of ADHD	Age	Gender	Brain MRI Finding	
				Normal	Abnormal region
1	HD/I	24	Female	*	-
2	HD/I	49	Male	*	-
3	HD/I	20	Male	*	-
4	AD	35	Male	-	Sub cortex
5	HD/I	45	Female	*	
6	HD/I	23	Female	*	
7	Combine	32	Female	-	Spelenium
8	Combine	19	Female	-	Pineal Gland
9	AD	25	Female	-	Retro cerebellar Archanoid
10	HD/I	20	Male	*	-
11	HD/I	21	Female	*	-
12	HD/I	19	Male	*	-
13	HD/I	20	Male	*	-
14	AD	41	Male	*	Frontal &Temporal Lobes
15	AD	21	Female	-	Frontal Lobe

HD/I: Hyperactivity/Impulsivity Disorder

AD: Attention Deficit Disorder

Combine: Attention Deficit/Hyperactivity Disorder

## 4. Discussion

According to the findings of the present study about 40% of adults showing ADHD symptoms suffer from a type of abnormality in the cortical sections or other parts of the brain (such as cerebellum, spelenium and pineal gland). The outstanding point of this research is that the MRI results for adults with hyperactivity subtype do not reflect any brain abnormality. However, all the cases with abnormal MRI show inattentive or combined symptoms of ADHD. The current results are somewhat similar to the results of resent studies conducted on children with ADHD (Batty et al., 2010; Carmona et al., 2009; Carmona et al., 2005; Cortese, 2012; Cubillo et al., 2012; Mackie et al., 2007; Rubia, 2011; Shaw et al., 2006). A review study by Cubillo et al. (2012) revealed that children and adults with ADHD are impaired in both cool and hot executive functions. These abnormalities in cognitive control are mediated by abnormalities in lateral inferior and dorsolateral prefrontal as well as some medial frontal regions and their regional interconnections with striatal, cerebellar, and parieto-temporal areas. The findings from imaging studies in adult ADHD suggests that the structural and functional brain abnormalities observed in children with ADHD persist into adult ADHD.

In neurocognitive approaches and models for ADHD, deficit in one executive function (i.e., inhibition) is considered to be the core of the disorder (Barkley, 1997). Individuals suffering from ADHD experience, (a), cognitive and attention, (b) emotional, and (c), motor abnormalities. Abnormalities in response inhibition, working memory, problem solving, planning, awareness, attention, and flexibility are the most important problems related to cognition and attention (Pasini & D'Agati, 2009; Sergeant et al., 2003). Schneider et al. (2006) emphasized on the role of cerebello-(thalamo-)-striato-cortical network in ADHD pathology from childhood to adulthood. They found changes in the architecture and function of prefrontal cortex and cerebellum in ADHD patients. Also, dysfunction of basal ganglia seems to be a consistent finding in childhood and adulthood ADHD. These findings confirm dysregulation of fronto-striatal network.

The frontal lobes, especially the prefrontal cortex, have several important functional parts that are associated with ADHD. Orbitofrontal lesions are accompanied by lack of inhibition and inability to control impulses; dorsolateral lesions are accompanied by malfunction of working memory and difficulty in planning; and mesial prefrontal lesions are accompanied by slow self-motivated behaviors (Seidman, Valera, & Makris, 2005).

Emotional abnormalities include motivational problems (Nigg & Casey, 2005), motor problems, difficulty in writing and motor coordination, delay in showing motor indicators, and clumsiness (Piek, Pitcher, & Hay, 1999) are observed in ADHD patients. Motor problems are largely related to structural or functional abnormalities in basal ganglia and the cerebellum (Pasini & D'Agati, 2009). Although, it is usually assume that the cerebellum is involved in motor control, but recent studies have revealed that cognitive and emotional processes are related to the cerebellum (Giedd et al., 2001; Hill et al., 2003; Schmahmann & Sherman, 1998). Mostofsky et al. (1998) argued that the cerebellum plays a role in cognition (executive function). They performed a study and found that size of the posterior vermis was significantly decreased in males with ADHD. This finding suggests that dysfunction within this region of the cerebellum may underlie clinical deficits seen in individuals with ADHD. A recent study by Stoodley et al. (2012) revealed that the cerebellar functional topography reflected the involvement of different cerebro-cerebellar circuits depending on the demands of the task being performed; motor tasks activated sensorimotor cortices along with contralateral cerebellar lobules IV-V and VIII, whereas cognitive tasks engaged prefrontal and parietal cortices along with cerebellar lobules VI and VII. Thus, cerebellum may have an important role in both motor and cognitive tasks. Also, It is suggested that in individuals with ADHD, the corpus callosum has abnormalities (Hill et al., 2003). These abnormalities often involve the posterior regions of the temporal and parietal cortexes in the spelenium. In addition, Damage to the left orbitofrontal region is reflected by emotional signs in adult ADHD cases (Hesslinger et al., 2002).

In sum, although different brain abnormalities have been reported to be associated with ADHD, and our findings support these reports to some extent, but none of the hyperactive participants showed an abnormality that could be traced by MRI. Nonetheless, this finding does not deny the existence of any brain abnormality in this group, because other abnormalities such as dysfunction of neurons and neurotransmitters which cannot be precisely assessed by MRI, accompany ADHD. Also, a strong relationship between neurotransmiter systems with ADHD has been reported in literature; including the dopamine system (David Viggiano et al., 2004), the NE system, cholinergic, and serotonin systems (Russell et al., 2000; Davide Viggiano et al., 2003; Adriani et al., 2003). It is said that some toxins that cause hyperDArgic function are correlated to changes in the cerebellar vermis (Ferguson & Cada, 2003). Moreover, the present study shows that the pineal gland may be abnormal in ADHD patients. The pineal gland has an effect on the dopaminergic system. Lavialle et al. (2010) reported an association between the drop in melatonin in the pineal gland and the increase in DA in the striatum and nucleus accumbens.

Finally, any structural damage to the brain that disturbs cognitive processes such as memorization, attention, inhibition, and brain circuits associated with reward and punishment, motivation and motion can contribute to the induction of ADHD. Clearly, proper understanding of results reflecting brain abnormalities becomes possible when the results are combined and merged with information on ADHD symptoms and the results of the studies conducted on the method and impact of treatments and accompanying disorders. Therefore, it is recommended to study different neuro-physiopathological, developmental, and genetic aspects of the etiology of ADHD.

Given high comorbidity of ADHD with a lot of other disorders, it is very difficult to find adult people with pure ADHD. For this reason, the study was conducted on a small sample size and this is a major limitation of the present study. These results therefore need to be interpreted with caution and more research on this topic needs to be undertaken before the association between ADHD subtypes and brain structural abnormalities is more clearly understood.
